# Paroxysmal Nonkinesigenic Dyskinesia with Tremor

**DOI:** 10.1155/2013/927587

**Published:** 2013-09-21

**Authors:** Robert Fekete

**Affiliations:** Department of Neurology, New York Medical College, Munger Pavilion, 4th Floor, 40 Sunshine Cottage Road, Valhalla, NY 10595, USA

## Abstract

*Introduction*. Paroxysmal nonkinesigenic dyskinesia (PNKD) consists of episodes of chorea, athetosis, or dystonia which are not triggered by movement, with complete remission between episodes. A case of genetically confirmed PNKD with simultaneous tremor has not been previously reported. *Case Report*. The patient is an 86-year-old right-handed female who presented with episodic stiffness, with onset at age 9. Attacks have a prodrome of difficulty in speaking, followed by abnormal sensation in extremities. Episodes consist of dystonia of trunk associated with upper and lower extremity chorea. There is complete resolution between attacks except for persistent mild head tremor and action tremor of both extremities. Attack frequency and duration as well as tremor amplitude escalated two and a half years ago, in correlation with development of breast carcinoma. Episodes improved after successful cancer treatment, but higher amplitude tremor persisted. There is an autosomal dominant family history of similar episodes but not tremor. Genetic diagnosis was confirmed via A7V mutation of the myofibrillogenesis regulator (*MR-1*) gene. *Conclusion*. Exacerbation due to another medical or psychiatric condition should be considered if there is unexpected deterioration in episode frequency or length. PNKD due to MR-1 mutation may exist even in the presence of action tremor.

## 1. Introduction

Paroxysmal nonkinesigenic dyskinesia (PNKD) consists of episodes of chorea, athetosis, or dystonia which are not triggered by movement, with complete remission between episodes [1]. Other names for this disorder include paroxysmal dystonic choreoathetosis (PDC), familial paroxysmal dyskinesia type 1 (FPD1), dystonia 8 (DYT8), and Mount-Reback syndrome. Mount and Reback used the term familial paroxysmal choreoathetosis in the first case description of PNKD in 1941. A case of genetically confirmed PNKD with simultaneous tremor has not been previously reported.

## 2. Case Report

The patient is an 86-year-old right-handed female who presents for evaluation of tremor and episodic stiffness. She had attacks of muscle stiffness since about age 9. Attacks have a prodrome of tongue heaviness and difficulty in speaking, followed by abnormal sensation in bilateral upper extremities. She remains fully conscious during the episodes. The actual episodes consist of dystonia of trunk associated with unilateral or bilateral upper and lower extremity chorea. About a quarter of the attacks are unilateral. There is complete resolution of symptoms between attacks except for persistent mild horizontal and vertical head tremor as well as action tremor of both extremities, also with onset at age 9. Hence she was able to play baseball and basketball at this age. There is an extensive family history of similar episodes but not tremor inherited in autosomal dominant fashion.

Psychological stress can trigger episodes, as a few have occurred just after medical appointments and phlebotomy. Caffeine exacerbates her condition. She refuses to drink coffee and only rarely drinks tea, both of which can cause attacks. She is able to handle a half a cup of hot chocolate with milk each morning. She does not drink alcohol. Surprisingly, evening diazepam did not prevent episodes and daytime as needed diazepam did not abort episodes. She will try clonazepam as that was reported to be more effective in PNKD [1].

The frequency of attacks was about once a month, but two and a half years ago it has escalated to once a day. Duration of attacks increased from half an hour to two hours. In addition, amplitude of action tremor of the hands as well as persistent horizontal and vertical head tremor worsened at that time. Two years ago she was diagnosed with breast cancer (3.3 cm Grade III infiltrative ductal carcinoma, Her2Neu positive, negative lymph node biopsy), which was treated with excision and localized radiation. Her episode frequency and length slowly returned towards baseline after treatment was completed.

In addition, she believes that her current exercise regimen utilizing 1 kg weights helps reduce frequency of attacks. This is in stark contrast to paroxysmal exercise induced dystonia (PED) and paroxysmal kinesigenic choreoathetosis/dyskinesia (PKC/PKD), which are episodic disorders triggered by exercise and sudden movements, respectively [2]. 

## 3. Discussion

The myofibrillogenesis regulator (*MR-1*) gene on chromosome 2q35 was tested. She was identified to have a nucleotide 20 C > T transition which corresponds to codon 7 alanine to valine substitution, abbreviated A7V. This as well as another reported mutation, A9V, disrupts the amino terminal alpha helix of the resulting protein [3]. The MR-1L isoform is expressed in the brain and localizes to the cell membrane [4]. The *MR-1* gene product was found to share homology with the hydroxyacylglutathione hydrolase (HAGH)/glyoxalase II gene via a similar but not identical *β*-lactamase domain. Methylglyoxal (pyruvaldehyde) is produced during glycolysis and is also present in coffee, tea, and alcoholic beverages. HAGH functions in a pathway to detoxify methylglyoxal [4]. Coffee contains about 76 *μ*g of methylglyoxal per serving compared to cocoa at 4.9 *μ*g per serving [5]. Her ability to tolerate cocoa as opposed to coffee can be explained by lower methylglyoxal content of cocoa, but the effect of lower caffeine content cannot be ruled out until the biochemical mechanism is precisely elucidated.

Antibody studies to rule out paraneoplastic syndrome at the onset of tremor exacerbation were unfortunately not performed, but the initial tremor presentation at age 9 is not due to paraneoplastic syndrome. Paroxysmal head tremor has been reported with *CACNA1A* mutation [6] but in conjunction with persistent cerebellar features as opposed to PNKD. The onset of the head tremor in that case was at 21 years old as opposed to age 9 in our case. Tremor exacerbation in this case is unlikely to be a drug effect as she was not treated with chemotherapy for her cancer.

This case demonstrates typical features of PNKD due to *MR-1* mutation: onset in infancy or early childhood, exacerbation by stress or caffeinated beverages, attacks of dystonia and/or chorea affecting the limbs or trunk, attack duration between 10 minutes and 1 hour, and family history of similar episodic disorder [1]. Bruno et al. propose normal neurological examination between attacks as a criterion for gene positive PNKD [1], but in this case action tremor of hands and head tremor persist between episodes. The presence of tremor may have been coincidental, but it is instructive to consider the possibility of PNKD even in the face of an abnormal neurological examination between episodes. It is also possible that the tremor is dystonic and may be related to the process causing paroxysmal dystonia and chorea.

## 4. Conclusion

Exacerbation due to another medical or psychiatric condition should be considered if there is unexpected deterioration in episode frequency or length. PNKD due to *MR-1* mutation may exist even in the presence of action tremor.
